# General Anesthesia or Spinal Anesthesia and Serum Endocan Release After Surgery: A Prospective Observational Study

**DOI:** 10.3390/jcm14228076

**Published:** 2025-11-14

**Authors:** Ergun Gunduz, Sinem Durmus, Naile Fevziye Misirlioglu, Oguzhan Cucu, Seyma Dumur, Bagnu Dundar, Hafize Uzun

**Affiliations:** 1 Department of Anaesthesiology and Intensive Care, Faculty of Medicine, Istanbul Atlas University, 34408 Istanbul, Turkey; 2 Department of Medical Biochemistry, Faculty of Medicine, Izmir Katip Celebi University, 35620 Izmir, Turkey; durmus.sinem@gmail.com; 3 Department of Medical Biochemistry, Faculty of Medicine, Istanbul Atlas University, 34408 Istanbul, Turkey; nailemisirlioglu@gmail.com (N.F.M.); seyma.dumur@atlas.edu.tr (S.D.); bagnu.dundar@atlas.edu.tr (B.D.); huzun59@hotmail.com (H.U.); 4 Department of Anaesthesiology and Intensive Care, Medicana Zincirlikuyu Hospital, 34394 Istanbul, Turkey; oguzhancucu@medicana.com.tr

**Keywords:** endocan, general anesthesia, spinal anesthesia, endothelial dysfunction, surgical patients

## Abstract

**Background/Objectives**: Awake procedures performed under spinal anesthesia (SA) have been associated with reduced hospitalization, costs, and postoperative complications compared with general anesthesia (GA). Endocan, an endothelial cell-specific proteoglycan, serves as a biomarker of endothelial activation and vascular dysfunction and may reflect the differential vascular and immunomodulatory effects of anesthetic techniques. This prospective observational study aimed to compare perioperative changes in circulating endocan levels between patients undergoing surgery under GA and SA. **Methods**: Eighty adult patients (aged 18–65 years, ASA I–II) scheduled for elective surgery were included and assigned to GA (*n* = 42) or SA (*n* = 38) based on standard clinical indications. Serum endocan levels were measured preoperatively, at 6 h, and at 24 h postoperatively using an ELISA assay. **Results**: In the GA group, endocan levels increased significantly from baseline (304.5 ± 80.7 pg/mL) to 6 h (511.5 ± 88.7 pg/mL, *p* < 0.001), and although partially decreased by 24 h (427.5 ± 87.9 pg/mL, *p* < 0.001), remained above baseline. In the SA group, endocan rose from baseline (320.7 ± 72.5 pg/mL) to 6 h (415.2 ± 79.5 pg/mL, *p* < 0.001) but returned near baseline at 24 h (352.6 ± 84.7 pg/mL, *p* = 0.233). **Conclusions**: These findings suggest that while surgery induces endothelial activation in both groups, GA is associated with a more sustained endothelial response than SA.

## 1. Introduction

Awake procedures performed under spinal (SA) or epidural (EA) anesthesia represent a viable alternative to general anesthesia (GA) in various surgical disciplines, including orthopedic, cardiothoracic, and neurosurgery. Compared with GA, awake surgery has been associated with favorable outcomes such as shorter hospitalization, lower healthcare costs, and reduced postoperative complications [1,2,3,4,5,6].

Endocan, also known as endothelial cell-specific molecule-1 (ESM-1), is a dermatan sulfate proteoglycan secreted by vascular endothelial cells and serves as a sensitive biomarker of endothelial activation and dysfunction [7]. It plays a key role in regulating angiogenesis, vascular permeability, and inflammatory processes [8]. Elevated serum endocan concentrations have been reported in pathological conditions such as sepsis, malignancy, obesity, hypertension, and systemic infection, reflecting endothelial injury and inflammatory stress [8,9,10]. Recent studies have further underscored its diagnostic and prognostic potential in endothelial dysfunction and cardiovascular disease, highlighting its role as a promising biomarker for vascular inflammation and damage [9,10].

During the perioperative period, anesthetic agents can influence the inflammatory response, cellular and biochemical defense mechanisms, and molecular mediators, potentially contributing to endothelial and epithelial injury [11]. In open-heart surgery under cardiopulmonary bypass, cardioplegia has been shown to cause endothelial damage [12,13]. GA may induce hypotension through vasodilation, and certain anesthetic agents can also cause pulmonary vasodilation [14,15].

The primary goal of anesthesia is to maintain hemodynamic stability while minimizing the adverse effects of anesthetic drugs, such as vasodilation, myocardial depression, and increased pulmonary vascular resistance. The objective of this study was to evaluate whether GA or SA results in significant changes in circulating endocan levels. By assessing this biomarker, which reflects endothelial activation and dysfunction, we aim to gain insight into the potential immunomodulatory and vascular effects of different anesthetic techniques. Understanding these effects may help clarify the relationship between anesthesia type, endothelial function, and perioperative inflammatory responses, ultimately contributing to improved patient management and safety.

This investigation also addresses the current gap in the literature regarding perioperative endothelial dynamics under distinct anesthesia modalities, an area not yet fully characterized in recent biomarker-based studies.

## 2. Materials and Methods

The study protocol received approval from the Ethics Committee of Istanbul Atlas University Medical Faculty (Approval number: E-22686390-050.99-60898; Date: 17 February 2025). The study was conducted in accordance with the principles outlined in the Declaration of Helsinki. All participants provided informed consent before participating in the study. Reporting of this observational study complies with the STROBE guidelines, and a completed checklist is provided as a Supplementary File. All participants provided informed consent before participating in the study.

### 2.1. Patient Selection

This study was a prospective observational comparative study evaluating serum endocan levels in patients undergoing elective surgery. Patients received either general or SA as part of standard clinical care. No randomization or investigator-directed allocation was performed. The study included 80 consecutive patients who underwent elective surgery at Istanbul Atlas University, Faculty of Medicine, Department of Anesthesiology. Patients were divided into two groups according to the anesthesia technique: the GA group (*n* = 40) and the SA group (*n* = 38).

The choice of anesthesia was based on patient preference whenever feasible, taking into account anesthetist recommendations and institutional protocols. Patients with contraindications to one of the techniques received the alternative anesthesia as determined by the attending anesthetist.

Inclusion criteria:

(i) Patients aged 18–65 years, (ii) ASA physical status I–II, (iii) Undergoing elective surgery for any indication.

Exclusion criteria:

(i) Presence of sepsis or active infection, (ii) Diagnosis of lung, breast, liver, kidney, or brain cancer, (iii) Obesity, (iv) Pregnancy, (v) Pediatric patients, (vi) ASA physical status III–IV, (vii) Advanced cardiac disease, (viii) Trauma patients, (ix) Emergency surgical cases.

### 2.2. Data Collection

Baseline demographic characteristics (age, sex, BMI) and clinical parameters were recorded. Routine biochemical (glucose, urea, creatinine, AST, ALT, electrolytes) and hematological (hemoglobin, white blood cell count, platelet count, coagulation indices) tests were obtained preoperatively. Hemodynamic variables, including systolic blood pressure, diastolic blood pressure, heart rate, and oxygen saturation, were monitored throughout the perioperative period.

From all patients, a 2 mL blood sample was collected into a K3 EDTA tube (Vacuette; Greiner Bio-One, Kremsmünster, Austria) for a complete blood count (CBC) parameter, whereas a 3 mL blood sample was drawn into standard tubes for biochemical parameters. Venous blood samples were collected at three time: preoperative baseline, early postoperative period (within 6 h after surgery), and late postoperative period (at 24 h after surgery). Serum endocan concentrations were measured using a commercially available Human Endothelial Cell-Specific Molecule-1 (Endocan) ELISA Kit (BT LAB, Cat. No: E3160Hu, Shanghai, China; Standard Curve Range: 5–2000 pg/mL; Sensitivity: 2.56 pg/mL). According to the manufacturer’s specifications, the intra-assay coefficient of variation (CV) was <8% and the inter-assay CV was <10%. All tests were conducted following the manufacturer’s guidelines.

Serum samples for endocan measurement were obtained at three timepoints: preoperatively, at postoperative 6 h (postoperative early), and at postoperative 24 h (postoperative late). The postoperative timepoints were selected based on the expected kinetics of endothelial and inflammatory activation following surgical stress. Previous studies have demonstrated that serum endocan levels increase rapidly after endothelial injury, typically peaking within a few hours after tissue trauma. Therefore, the 6-h sample was intended to capture the early postoperative response. The 24-h sample was chosen to evaluate short-term recovery dynamics and potential normalization trends within the first postoperative day.

The results of the CBC were recorded using an automatic hematology analyzer (Sysmex XN-1000, Norderstedt, Germany). Routine biochemical blood parameters were measured with an automated analyzer (COBAS 8000, Roche, 2007, Tokyo, Japan).

### 2.3. Statistical Analysis

All statistical analyses were performed using JASP (version 26.0). Continuous variables were expressed as mean ± standard deviation (SD), and categorical variables as frequencies and percentages. Normality of distribution was assessed with the Shapiro–Wilk test and Q–Q plots. For continuous variables, independent samples *t*-tests were used to compare baseline characteristics between groups. For categorical variables, chi-square tests were applied to evaluate the association between anesthesia type and surgical procedure, medication use, and gender.

To assess changes in endocan levels across the perioperative period (preoperative, early postoperative, late postoperative), repeated measures ANOVA were conducted separately for the GA and SA groups. The assumption of sphericity was examined using Mauchly’s test, and Greenhouse–Geisser corrections were applied when necessary. Post hoc pairwise comparisons were performed with Bonferroni correction. Non-parametric Friedman tests were additionally reported for robustness. A two-tailed *p*-value < 0.05 was considered statistically significant.

To control potential confounding effects, multivariate linear regression analyses were conducted with postoperative early and late endocan levels as dependent variables. Independent variables included preoperative endocan level, anesthesia type, surgery duration, and age. Model fit was assessed using the coefficient of determination (R^2^) and the F-test for overall significance. Standardized regression coefficients (β) and associated *p*-values were reported for each predictor.

Sample size estimation was based on previously published plasma endocan data [10]. Using G*Power 3.1 for a repeated-measures design with two groups and two measurements, alpha = 0.05, power = 0.8, and effect size f = 0.28, the calculated sample size was 78. Considering a potential 10% data loss, the target recruitment was set at 86 patients. A total of 80 patients completed the study protocol, which was deemed sufficient to detect differences in perioperative endocan trajectories.

## 3. Results

A total of 80 patients were included, 42 receiving GA and 38 SA. Chi-square analysis revealed a significant association between surgical procedure and anesthesia type (χ^2^(21, *n* = 80) = 55.004, *p* < 0.001; Table 1). The groups were comparable in terms of BMI, glucose, urea, creatinine, liver enzymes, electrolytes, hematological parameters, and most vital signs (Table 2). However, surgery duration was significantly longer in the GA group compared to SA (205.60 ± 114.31 vs. 99.34 ± 38.40 min, *p* < 0.001). Hemoglobin levels were lower (*p* = 0.006), while systolic blood pressure was lower in the GA group compared to SA (*p* = 0.006). Oxygen saturation was slightly but significantly higher in the GA group (*p* = 0.026).

No significant association was found between medication use and anesthesia type (χ^2^(17, *n* = 80) = 21.005, *p* = 0.226). Gender was significantly associated with anesthesia type (χ^2^(2, *n* = 80) = 11.688, *p* = 0.003).

**Figure 1:**
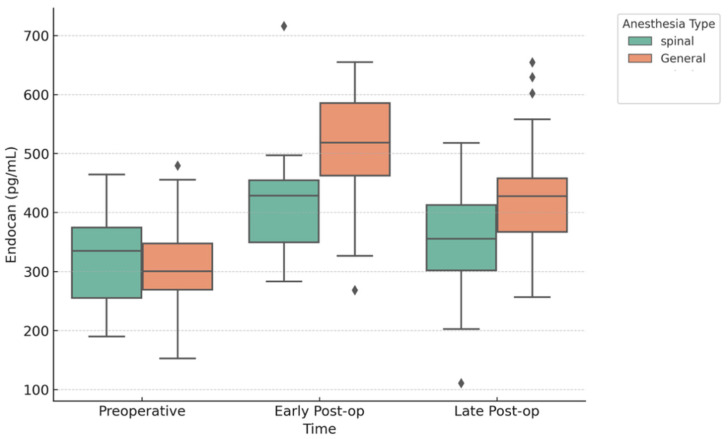
Distribution of endocan levels in patients undergoing GA and SA across the perioperative period (preoperative, early postoperative, late postoperative).

Figure 1 illustrates the distribution of endocan levels across the perioperative period by anesthesia type. In patients receiving GA, a repeated-measures ANOVA demonstrated a significant effect of time on circulating endocan levels (F(2,82) = 50.91, *p* < 0.001). Mauchly’s test indicated that the assumption of sphericity was met, *W* = 0.996, χ^2^(2) = 0.164, *p* = 0.921. Consistent with this, a Friedman test confirmed the robustness of the findings, χ^2^(2) = 45.19, *p* < 0.001, Kendall’s W = 0.54, suggesting a large effect size. Post hoc analyses (Table 3) revealed that endocan levels increased markedly from the preoperative (304.50 ± 80.67 pg/mL) to the early postoperative period (511.50 ± 88.73 pg/mL; *p* < 0.001, *d* = −2.41), followed by a partial but significant decrease in the late postoperative period (427.5 ± 87.9 pg/mL; *p* < 0.001, *d* = −1.43). Nevertheless, endocan levels remained significantly higher at the late postoperative stage compared to baseline.

In patients receiving SA, repeated-measures ANOVA indicated a significant effect of time on endocan levels, F(2,74) = 14.90, *p* < 0.001. Mauchly’s test confirmed that the assumption of sphericity was not violated, *W* = 0.931, χ^2^(2) = 2.567, *p* = 0.277. Consistently, a Friedman test also supported these findings, χ^2^(2) = 16.47, *p* < 0.001, Kendall’s W = 0.22, reflecting a moderate effect size. Post hoc pairwise comparisons (Table 4) showed that endocan levels significantly increased from the preoperative (320.7 ± 72.5 pg/mL) to the early postoperative period (415.2 ± 79.5 pg/mL; *p* < 0.001, *d* = −1.20). Although a decrease was observed in the late postoperative period (352.6 ± 84.7 pg/mL), the difference compared to baseline did not reach statistical significance (*p* = 0.233). However, endocan levels were significantly reduced in the late postoperative period compared to the early postoperative peak (*p* = 0.008, *d* = 0.79).

The observed effect sizes (Cohen’s *d*) indicated that changes in serum endocan levels were large in magnitude, particularly in the general anesthesia (GA) group. In this group, the decrease from preoperative to early postoperative levels (*d* = 2.41) represent a very large effect, suggesting a pronounced and biologically relevant increase in endothelial activation shortly after surgery. The moderate-to-large difference observed between early and late postoperative values (*d* = 0.98) indicates partial recovery of endothelial function within the first 24 h. In the spinal anesthesia (SA) group, effect sizes were smaller (preoperative vs. early postoperative, *d* = 1.20; early vs. late, *d* = 0.79), reflecting a less pronounced and more transient endothelial response compared with GA. These magnitudes support the clinical relevance of anesthesia-related differences in endothelial activation.

Multivariate linear regression was performed to evaluate the independent association between anesthesia type and postoperative serum endocan levels, after adjusting for potential confounders (preoperative endocan, surgery duration, and age). For early postoperative endocan, the overall model was significant (F(4,75) = 6.95, *p* < 0.001; adjusted R^2^ = 0.23). Among the covariates, only anesthesia type remained a significant independent predictor (β = −98.9, *p* < 0.001). For late postoperative endocan, the model also reached statistical significance (F(4,75) = 4.22, *p* = 0.004; adjusted R^2^ = 0.14). Similarly, only anesthesia type was independently associated with post-operative late endocan (β = −72.6, *p* = 0.002). Neither surgery duration, age, nor preoperative endocan levels showed a significant effect in either model (Table 5).

## 4. Discussion

In this study, we investigated the perioperative changes in endocan levels in patients undergoing surgery under GA or SA. To the best of our knowledge, this is the first study to directly compare perioperative endocan dynamics between GA and SA, providing novel insights into the vascular consequences of different anesthetic approaches. Our findings demonstrate that endocan, a marker of endothelial activation, increases significantly during the perioperative period, with a more pronounced and sustained elevation observed in patients receiving GA compared to SA. These results suggest that anesthesia type may influence both the magnitude and duration of endothelial response, reflecting differences in systemic inflammatory activation. Understanding these variations could have important implications for perioperative management and prevention of complications related to endothelial dysfunction.

These findings align with previous studies indicating that GA can induce a more sustained inflammatory response compared to SA. For instance, Honca et al. [16] observed that GA was associated with increased levels of endothelial adhesion molecules in fetal circulation during cesarean section, reflecting heightened endothelial activation. Similarly, Vosoughian et al. [17] reported higher postoperative levels of pro-inflammatory cytokines such as IL-6 and TNF-α in patients undergoing cesarean section under GA compared to SA. Thus, SA may provide a more favorable inflammatory profile in certain surgical contexts.

The differential endothelial response between GA and SA may be attributed to their distinct physiological and immunological mechanisms. GA commonly involves endotracheal intubation, mechanical ventilation, and exposure to volatile or intravenous anesthetic agents, which can activate the sympathetic nervous system, induce oxidative stress, and increase circulating stress hormones such as catecholamines and cortisol. These factors can disrupt endothelial homeostasis, leading to sustained endothelial activation and increased vascular permeability. In contrast, SA produces a sympathetic blockade that blunts the neuroendocrine stress response, reduces catecholamine release, and improves microcirculatory perfusion, explaining the transient and less pronounced endocan elevation observed. Differences in intraoperative oxygen delivery, inflammatory mediator release, and hemodynamic stability may further contribute to distinct endocan kinetics.

GA may also transiently affect innate immune function, whereas SA avoids these systemic effects, resulting in a more favorable endothelial response. The return of endocan levels to baseline in the SA group suggests transient endothelial activation, potentially reducing the risk of postoperative complications associated with prolonged endothelial stress [16,18,19].

Consistent with Bouglé et al. [20], who reported that postoperative endocan levels correlate with the duration of norepinephrine support after coronary artery bypass surgery, our findings indicate that GA induces a more sustained elevation of endocan compared to SA. Dogdus et al. [21] and Ye et al. [22] similarly demonstrated that elevated endocan predicts endothelial dysfunction and microvascular impairment. Taken together, these data support endocan as a sensitive perioperative marker of endothelial function, with kinetics influenced by anesthesia type.

Scherpereel et al. [23] first identified endocan as a marker of endothelial activation in sepsis. Subsequent studies have reinforced its utility as a dynamic biomarker of endothelial stress [24,25,26,27]. In addition, endocan promotes the expression of intercellular adhesion molecule-1 (ICAM-1), vascular cell adhesion molecule-1 (VCAM-1), and E-selectin, enhancing leukocyte-endothelial adhesion and facilitating inflammatory cell migration. VCAM-1 contributes to microvascular activation and arterial dysfunction, while intermittent hypoxia can upregulate endocan via the HIF-1α/VEGF pathway, promoting ICAM-1/VCAM-1 expression and monocyte-endothelial adhesion [28]. These mechanisms further support the role of endocan as a sensitive marker of endothelial activation and vascular inflammation.

Surgical duration differed significantly between groups, which may independently influence systemic inflammatory responses and endothelial activation. While multivariate analyses adjusting for surgical duration confirmed that anesthesia type remained an independent predictor of endocan levels, residual contributions from procedural factors—including fluid therapy, anesthetic agents, and perioperative drug use—cannot be fully excluded. Other perioperative variables, such as blood loss or vasopressor use, were not systematically recorded and could also impact endothelial responses.

Due to the observational design, causal relationships between anesthesia type and perioperative endothelial function cannot be definitively established. While the data suggest an association, the contribution of unmeasured confounders cannot be excluded.

Although statistically significant differences were observed, the clinical relevance of perioperative endocan changes remains to be fully determined. There is no universally accepted threshold for a clinically meaningful increase; however, sustained elevations have been associated with endothelial dysfunction, microvascular impairment, and adverse outcomes. Further studies with larger cohorts and longer follow-up are warranted.

This study has several strengths, including a prospective design with well-defined perioperative time points for endocan measurement, direct comparison between GA and SA, and comprehensive assessment of clinical, laboratory, and hemodynamic parameters. Limitations include the observational design, small sample size, single-center setting, short-term follow-up, lack of additional inflammatory biomarkers, and variability in surgical types and durations, which may limit generalizability and contribute to residual confounding despite multivariate adjustments.

In conclusion, perioperative serum endocan levels increase significantly following surgery, with GA associated with a more sustained elevation and SA showing a transient response. While these findings suggest that anesthesia type may influence endothelial activation, causation cannot be inferred from this observational study. Larger randomized multicenter studies with longer follow-up and additional biomarkers are needed to confirm these results and determine their clinical significance.

In conclusion, perioperative serum endocan levels increased significantly following surgery, with GA associated with a more sustained elevation and SA showing a transient response. These findings highlight endocan as a sensitive biomarker of perioperative endothelial activation and suggest that anesthesia type may modulate vascular and inflammatory responses. However, causation cannot be inferred from this observational study. Future multicenter randomized trials with larger cohorts, extended follow-up, and inclusion of additional endothelial and inflammatory biomarkers are warranted to confirm these findings and to better define the clinical relevance and potential applications of endocan in perioperative medicine.

## Figures and Tables

**Table 1:** Distribution of Anesthesia Type by Surgical Procedure.

Surgical Procedure	GA *n* (%)	SA *n* (%)	Total *n* (%)
Arthroscopy	1 (2.4%)	4 (10.5%)	5 (6.2%)
Knee prosthesis	1 (2.4%)	5 (13.2%)	6 (7.5%)
Inguinal hernia	2 (4.8%)	2 (5.3%)	4 (5.0%)
Hip prosthesis	0 (0.0%)	1 (2.6%)	1 (1.2%)
Cystectomy	1 (2.4%)	0 (0.0%)	1 (1.2%)
Lumbar disc herniation	4 (9.5%)	0 (0.0%)	4 (5.0%)
Liposuction	9 (21.4%)	0 (0.0%)	9 (11.2%)
Breast prosthesis	1 (2.4%)	0 (0.0%)	1 (1.2%)
Meniscus surgery	0 (0.0%)	2 (5.3%)	2 (2.5%)
Osteomyelitis	0 (0.0%)	1 (2.6%)	1 (1.2%)
Ovarian cystectomy	1 (2.4%)	0 (0.0%)	1 (1.2%)
Pilonidal sinus	0 (0.0%)	3 (7.9%)	3 (3.8%)
Rhinoplasty	9 (21.4%)	0 (0.0%)	9 (11.2%)
Cystocele repair	1 (2.4%)	2 (5.3%)	3 (3.8%)
Stapedectomy	1 (2.4%)	0 (0.0%)	1 (1.2%)
TUR (transurethral)	0 (0.0%)	2 (5.3%)	2 (2.5%)
Umbilical hernia	2 (4.8%)	0 (0.0%)	2 (2.5%)
Ureterorenoscopy (URS)	1 (2.4%)	1 (2.6%)	2 (2.5%)
Varicocele	2 (4.8%)	8 (21.1%)	10 (12.5%)
Varicose vein surgery	1 (2.4%)	5 (13.2%)	6 (7.5%)
Facelift	5 (11.9%)	0 (0.0%)	5 (6.2%)
ACL reconstruction	0 (0.0%)	2 (5.3%)	2 (2.5%)
Total	42 (100%)	38 (100%)	80 (100%)

Note. Chi-square test indicated a significant association between surgical procedure and type of anesthesia, χ^2^(21, *n* = 80) = 55.004, *p* = 7.059 × 10^−5^.

**Table 2:** Baseline Characteristics and Laboratory Parameters of Patients Undergoing General vs. Spinal Anesthesia.

Variable	GA (Mean ± SD)	SA (Mean ± SD)	*p*-Value
	(*n* = 42)	(*n* = 38)	
Duration of surgery (min)	205.60 ± 114.31	99.34 ± 38.40	5.578 × 10^−7^
BMI	43.97 ± 5.75	45.32 ± 4.56	0.249
Glucose (mg/dL)	95.50 ± 16.84	102.24 ± 22.66	0.133
Urea (mg/dL)	15.60 ± 3.84	17.36 ± 5.01	0.081
Creatinine (mg/dL)	0.76 ± 0.14	0.82 ± 0.16	0.073
AST (U/L)	22.60 ± 11.79	24.13 ± 11.76	0.562
ALT (U/L)	24.24 ± 8.72	23.71 ± 7.08	0.769
Sodium (Na, mmol/L)	138.83 ± 2.49	138.63 ± 2.55	0.721
Potassium (K, mmol/L)	4.02 ± 0.25	4.03 ± 0.21	0.783
Hemoglobin (Hb, g/dL)	13.32 ± 1.54	14.43 ± 1.99	0.006
White Blood Cells (WBC, ×10^3^/µL)	7.48 ± 1.99	7.94 ± 2.11	0.316
Platelets (PLT, ×10^3^/µL)	262.50 ± 60.65	249.32 ± 54.03	0.310
Neutrophils (%)	54.82 ± 9.17	55.53 ± 9.28	0.732
Lymphocytes (%)	39.99 ± 8.47	33.01 ± 8.69	0.278
INR	0.97 ± 0.06	1.19 ± 1.49	0.336
Pulse rate (beats/min)	76.93 ± 7.81	80.26 ± 9.05	0.081
Oxygen saturation (SpO_2_, %)	98.81 ± 0.71	98.45 ± 0.72	0.026
Systolic BP (mmHg)	121.48 ± 9.89	127.47 ± 8.82	0.006
Diastolic BP (mmHg)	71.69 ± 8.81	74.76 ± 9.37	0.135
Preoperative Endocan	304.50 ± 80.67	320.74 ± 72.50	0.349
Post-op Early Endocan	511.50 ± 88.73	415.18 ± 79.53	2.403 × 10^−6^
Post-op Late Endocan	427.50 ± 87.87	352.58 ± 84.67	2.209 × 10^−4^

BMI, Body Mass Index; AST, Aspartate Aminotransferase; ALT, Alanine Aminotransferase; INR, International Normalized Ratio; BP, Blood Pressure.

**Table 3:** Changes in Endocan Levels (pg/mL) Across the Perioperative Period in the GA Group.

Comparison	Mean Difference	SE	df	t	Cohen’s *d*	*p*
Preoperative vs. Post-op Early	−207.00	19.97	41	−10.37	−2.41	1.521 × 10^−12^
Preoperative vs. Post-op Late	−123.00	20.89	41	−5.89	−1.43	1.874 × 10^−6^
Post-op Early vs. Late	84.00	21.03	41	3.99	0.98	7.890 × 10^−4^

Note: *p*-values adjusted using Bonferroni correction. Effect size (Cohen’s *d*) values are interpreted as small (0.2), medium (0.5), and large (0.8). The observed values, particularly in the GA group, correspond to large to very large effects, indicating biologically meaningful postoperative changes in endothelial activation.

**Table 4:** Changes in Endocan Levels (pg/mL) Across the Perioperative Period in the SA Group.

Comparison	Mean Difference	SE	df	t	Cohen’s *d*	*p*
Preoperative vs. Post-op Early	−94.45	15.51	37	−6.09	−1.20	1.425 × 10^−6^
Preoperative vs. Post-op Late	−31.84	17.55	37	−1.81	−0.40	0.233
Post-op Early vs. Late	62.61	19.53	37	3.21	0.79	0.008

Note, *p*-values adjusted using Bonferroni correction. Effect size (Cohen’s *d*) values are interpreted as small (0.2), medium (0.5), and large (0.8). The observed values, particularly in the GA group, correspond to large to very large effects, indicating biologically meaningful postoperative changes in endothelial activation.

**Table 5:** Multiple Linear Regression Analyses for Post-op Early and Late Endocan Levels.

Dependent Variable	Predictor	β (Unstandardized)	SE	β (Standardized)	t	*p*	Adjusted R^2^
Post-operative early Endocan	(Intercept)	579.469	67.609	—	8.571	<0.001	0.232
	Pre-operative endocan	−0.035	0.128	−0.027	−0.270	0.788	
	Anesthesia Type	−98.879	22.361	−0.513	−4.422	<0.001	
	Surgery Duration	−0.025	0.112	−0.026	−0.220	0.826	
	Age	1.081	0.740	0.145	1.460	0.148	
Post-operative late Endocan	(Intercept)	569.196	69.161	—	8.230	<0.001	0.140
	Pre-operative endocan	−0.142	0.131	−0.116	−1.083	0.282	
	Anesthesia Type	−72.637	22.875	−0.390	−3.175	0.002	
	Surgery Duration	−0.003	0.115	−0.003	−0.023	0.981	
	Age	−0.587	0.757	−0.082	−0.775	0.441	

Note: Models adjusted for pre-operative endocan, surgery duration, and age. Only the anesthesia type was significantly associated with both postoperative early and late endocan levels.

## Data Availability

The data underlying this article are available in the article. If needed, please contact the corresponding author. The email address is ergun.gunduz@atlas.edu.tr.
